# Aerobic exercise for vasomotor menopausal symptoms: A cost-utility analysis based on the Active Women trial

**DOI:** 10.1371/journal.pone.0184328

**Published:** 2017-09-26

**Authors:** Ilias Goranitis, Leana Bellanca, Amanda J. Daley, Adele Thomas, Helen Stokes-Lampard, Andrea K. Roalfe, Sue Jowett

**Affiliations:** 1 Health Economics Unit, University of Birmingham, Birmingham, United Kingdom; 2 Primary Care Clinical Sciences, University of Birmingham, Birmingham, United Kingdom; 3 Office of the Dean, Higher Degree Research, Macquarie University, Sydney, NSW, Australia; University of Miami School of Medicine, UNITED STATES

## Abstract

**Objective:**

To compare the cost-utility of two exercise interventions relative to a control group for vasomotor menopausal symptoms.

**Design:**

Economic evaluation taking a UK National Health Service and Personal Social Services perspective and a societal perspective.

**Setting:**

Primary care.

**Population:**

Peri- and postmenopausal women who have not used hormone therapy in the past 3 months and experience ≥ 5 episodes of vasomotor symptoms daily.

**Methods:**

An individual and a social support-based exercise intervention were evaluated. The former *(Exercise-DVD)*, aimed to prompt exercise with purpose-designed DVD and written materials, whereas the latter *(Exercise-Social support)* with community exercise social support groups. Costs and outcomes associated with these interventions were compared to those of a control group, who could only have an exercise consultation. An incremental cost-utility analysis was undertaken using bootstrapping to account for the uncertainty around cost-effectiveness point-estimates.

**Main outcome measure:**

Cost per quality-adjusted life-year (QALY).

**Results:**

Data for 261 women were available for analysis. *Exercise-DVD* was the most expensive and least effective intervention. *Exercise-Social support* was £52 (CIs: £18 to £86) and £18 (CIs: -£68 to £105) more expensive per woman than the control group at 6 and 12 months post-randomisation and led to 0.006 (CIs: -0.002 to 0.014) and 0.013 (CIs: -0.01 to 0.036) more QALYs, resulting in an incremental cost-effectiveness ratio of £8,940 and £1,413 per QALY gained respectively. *Exercise-Social support* had 80%-90% probability of being cost-effective in the UK context. A societal perspective of analysis and a complete-case analysis led to similar findings.

**Conclusions:**

*Exercise-Social support* resulted in a small gain in health-related quality of life at a marginal additional cost in a context where broader wellbeing and long-term gains associated with exercise and social participation were not captured. Community exercise social support groups are very likely to be cost-effective in the management of vasomotor menopausal symptoms.

## Introduction

The hormonal changes during the menopausal transition in a woman’s life are commonly associated with a number of physical and psychological symptoms [1]. The most prevalent symptoms among peri- and postmenopausal women are hot flushes and night sweats, known as vasomotor menopausal symptoms. In the 2005 European Menopause Survey, 82% of postmenopausal women in the UK reported vasomotor symptoms over the past five years [2], and evidence suggests that 87% of these women experience symptoms on a daily basis, with 33% having more than ten episodes a day [3]. The adverse impact of these symptoms on health and wellbeing is well established [4–7].

Hormone therapy, with estrogen alone or in combination with progestogen, is considered to be the most effective treatment option for vasomotor menopausal symptoms [3]. However, evidence linking hormone therapy with increased risk of breast cancer, stroke, coronary heart disease, and thromboembolic disease [8–10], coupled with adverse side-effects and extensive negative media reports, have led women seeking alternative treatment options [11]. In the quest for alternative therapies, exercise has been commonly advocated. There are biological, psychological, and psychosocial explanations of why exercise may potentially be an effective treatment option for vasomotor symptoms [12], and some supporting evidence for a positive impact on many symptoms and health conditions typically associated with menopausal transition exists [13–15]. For example, exercise may help to stabilise the thermoregulatory centre of the brain through beta-endorphin production and contribute towards the improvement of mental wellbeing, sense of achievement and self-esteem.

The Scientific Advisory Committee of the Royal College of Obstetricians and Gynaecologists in the UK and their Patient Information Committee advise that regular sustained aerobic exercise may ameliorate vasomotor menopausal symptoms [16, 17]. However, the latest Cochrane review [18], concluded that there is lack of evidence showing that exercise is an effective treatment for these symptoms and called for further research. Since then, findings from a randomised controlled trial concluded that 3 months of moderate exercise does not alleviate vasomotor symptoms, but leads to statistically significant improvements in depressive symptoms, sleep quality and insomnia [19]. The Active Women trial assessed the effectiveness of a 6-month exercise intervention, with a further 6-month follow-up, and concluded that exercise reduces the frequency of vasomotor symptoms per week relative to a control group but the reduction is not statistically significant [20]. At the end of the trial, improvements were observed in a number of secondary outcomes, such as depressive symptoms and anxiety, sleep quality and sexual behaviour, but these improvements were also not significantly different between the exercise groups and the control group, but in some instances they were clinically meaningful.

Despite this debate on the clinical effectiveness of exercise, limited attention has been paid to the issue of cost-effectiveness. Economic evidence is commonly used in the United Kingdom and beyond to inform decision makers about the best use of scarce resources within the health care context. For women with vasomotor menopausal symptoms, the first economic evaluation was published in August 2015 and concluded that physical activity is cost-effective at 6 months follow-up, as the benefits of exercise were obtained at a very low cost [21]. This economic evaluation was published 3 years after the primary clinical study [22]. Another economic evaluation focusing on perimenopausal women is currently in progress [23], and more studies assessing the cost-effectiveness of exercise in the management of vasomotor symptoms are needed.

The lack of economic analyses in this context is perhaps attributed to the lack of comprehensive clinical evidence demonstrating a significant effect of exercise on the frequency or severity of vasomotor symptoms. In order to inform resource allocation decisions, in a comparative way across a range of health care interventions, economic analyses are not restricted to one clinical outcome only, such as the absolute reduction in the frequency of vasomotor symptoms, but aim to capture a wide set of outcomes through their impact on preference-based health-related quality of life [24].

Evidence that menopause impacts on health-related quality of life [25], and that exercise improves quality of life in menopausal women [26, 27], even in women with vasomotor menopausal symptoms [22, 28, 29], exists. Improvements in the frequency or severity of vasomotor symptoms, and equally improvements in depressive symptoms, anxiety, sleep quality and sexual behaviour, even though they may not always be found to be statistically significant in absolute terms, they may have a valuable net effect on a woman’s quality of life. It is these benefits and the opportunity cost associated with those benefits that matters in health care resource allocation decisions. This study aims to evaluate the cost-utility of an individual and a social version of an exercise intervention relative to a control group based on data from the Active Women trial.

## Methods

### Trial design and participants

Favourable ethical opinion for this study was granted by the West Midlands Research Ethics Committee in March 2010 (ref: 10/H1208/3).This is UK National Research Ethics Service (NRES) ethical approval, sought as required by the University of Birmingham.

The study presents cost-utility data from the Active Women’s trial. Information about the trial design and participants can be found in the published trial protocol [30], and the accompanying clinical paper [20]. In brief, women aged 48–57 years were contacted from 23 general practices across the West Midlands in the UK between April 2011 and September 2014. To be included in the trial, women had to be peri- or postmenopausal, experience ≥ 5 episodes of hot flushes and/or night sweats daily of any severity, and had not used hormone therapy in the past 3 months. To be eligible, women also had to be inactive according to the UK’s public health guidelines for physical activity, namely achieve less than 30 minutes of moderate intensity exercise, like brisk walking and cycling, on at least 5 days per week [31], and in a position to provide written informed consent and to communicate in English. Women were subsequently randomised to one of two 6-month exercise interventions or a control group on a 1:1:1 ratio, and were followed-up at 6 and 12 months post-randomisation. Information on the use of hormone therapy or other complementary and alternative medicine was recorded at baseline and follow-up periods.

The two exercise interventions aimed at achieving 30 minutes of moderate intensity aerobic exercise on at least 3 days a week during the first 3 months of the trial, and on 3–5 days a week during the following 3 months. Both interventions involved two 40–60 minute one-to-one consultations with a physical activity facilitator at a participant’s home. In one exercise intervention women were sent a purpose-designed DVD, a booklet, and five study leaflets, which were expected to act as regular reminders that would prompt exercise. This intervention will be referred to as *“Exercise-DVD”*. In the other intervention, women were invited to three exercise social support groups in their community lasting 75–90 minutes. These were aimed to act as social nudges by bringing women together to share experiences and support. This intervention will be referred to as *“Exercise-Social support”*. The control group involved the opportunity of an exercise consultation and women in this group were given a pedometer at the end of their involvement in the study.

### Resource use and costs

Resource use information was collected prospectively using self-completed postal questionnaires at 6 and 12 months post-randomisation. Resource use information was collected from both a National Health Service and Personal Social Services perspective (NHS/PSS) and a societal perspective. The NHS/PSS perspective included resource use relevant to contacts for vasomotor menopausal symptoms with general practitioners and practice nurses (at the surgery or out-of-hours telephone consultations), gynaecologists or other hospital doctors, and counsellors or psychologists. Information on relevant repeat prescriptions and free prescriptions was also recorded. The societal perspective additionally included resource use related to contacts with private therapists for any complementary and alternative medicines for vasomotor menopausal symptoms, time off work and other activities due to the menopausal symptoms, and out-of-pocket payments for relevant prescription and non-prescription medicines. Information on hospital admissions was captured in the questionnaire, but these were not related to vasomotor menopausal symptoms, and thus were excluded from the two costing perspectives.

Unit costs for public health and community services were obtained from national sources [32, 33], and are presented in Table 1. Mean medication costs for vasomotor menopausal symptoms’ were estimated based on specific dosage regimens for the ten most commonly prescribed medicines, and unit costs were obtained from the *British National Formulary* [34]. Costs related to the development and delivery of the exercise interventions were calculated using a standard micro-costing approach [35]. Productivity costs associated with paid and unpaid work were calculated using the human capital approach, based on average age- and gender-specific wage rates, and the proxy good approach respectively [36]. These costs were obtained from the Office for National Statistics (ONS) [37]. Private complementary and alternative therapies were costed on an individual basis using online available market prices. In the societal perspective, NHS/PSS costs were adjusted for value added tax [38], using data from the World Bank [39]. All unit costs were valued in UK pounds sterling for the financial year 2013/14.

**Table 1:** Resource use categories and associated unit costs (£, 2013/2014 prices).

Resource use	Unit cost	References
Consultation with general practitioner (per 17 minute contact)	66	PSSRU [32]
Out of hours telephone consultation with general practitioner (per 7.1 minute contact)	27	*Ibid*.
Consultation with practice nurse (per 15.5 minute contact)	13	*Ibid*.
Out of hours telephone consultation with practice nurse (per 15.5 minute contact)	10	*Ibid*.
Consultation with gynaecologist (per contact)	134	*Ibid*.
Consultation with a counsellor or psychologist (per 50 minute contact)	112	*Ibid*.
Physiotherapy (per 23.3 minute contact)	14	*Ibid*.
X-ray (per scan)	91	NHS reference cost [33]
Prescription medicines (per 6 months)	38	BNF [34]
Repeat prescription for HRT (per 3 months)	19	*Ibid*.
Repeat prescription without seeing the doctor (per prescription)	8	Department of Health [40]
Time off paid work (Full-time employed)	93	ONS [37]
Time off paid work (Part-time employed)	67	*Ibid*.
Time off unpaid work (per hour of housekeeping work)	8	*Ibid*.
Private therapy (mean estimate of patient-reported therapies)	51	Online market prices

### Health-related quality of life

Health-related quality of life was assessed based on participants’ responses to the SF-12 at baseline, 6 and 12 months follow-up. A preference-based index of health-related quality of life for use in the economic evaluation was generated by mapping the different response permutations to SF-12 onto the SF-6D health state classification [41]. Health state valuation for the SF-6D was undertaken from a representative sample of 611 members of the general public in the UK based on the standard gamble method and ranges from 0.345 to 1 (full health) [41, 42]. Quality-adjusted life-years (QALYs) for each woman were calculated using the area under the curve approach [43].

### Missing data

To maintain efficiency and avoid potential biasing of results by excluding participants with missing data [44, 45], multiple imputation for total costs, and health-related quality of life scores was applied using chained equations with predictive mean matching over 10 imputations [46]. For each imputed variable, model covariates with *p-value* ≤ 0.30 were selected using stepwise multiple linear regressions from age, BMI classification, menopause status, use of antidepressants or hormone therapy, frequency of vasomotor menopausal symptoms, group allocation, and baseline quality of life. To maintain within- and between-imputation variability, multiply imputed datasets were analysed individually [47], and cost-effectiveness estimates were derived according to Rubin’s rules [48].

### Cost-utility analysis

An incremental cost-utility analysis based on the outcome of cost per QALY was conducted from an NHS/PSS perspective [35], in line with recommended practices in the UK [49], and a societal perspective. Economic analyses were conducted on an intention-to-treat basis using the multiple imputed data. Due to the 12-month time-horizon, costs and outcomes were not discounted. The cost-utility of the two exercise interventions relative to the control group was assessed at 6 and 12 months post-randomisation. A complete-case analysis was also undertaken to explore the robustness of the study’s results.

Differences in mean costs and outcomes between comparators and 95% confidence intervals (CIs) for the main (multiple imputation) analysis were obtained according to Rubin’s rules [48], using multiple linear regression with robust standard errors based on Huber-White sandwich estimators. For the complete-case analysis, differences and 95% CIs were obtained using 1,000 bias-corrected and accelerated (BCa) bootstrap resamples [50]. To account for the uncertainty around cost-effectiveness point-estimates, non-parametric bootstrapping with multiple linear regression was used to generate a joined distribution of 5,000 incremental mean cost and outcome estimates for the different comparators [51, 52]. For this, covariate selection with *p-*value ≤ 0.30 was performed with stepwise regressions. Covariates for costs included age, BMI classification, menopause status, and frequency of symptoms. Covariates for QALYs additionally included baseline quality of life, inpatient stays, employment status, change in the use of antidepressants, and change in symptoms’ frequency [53]. The paired estimates from bootstrapping were used to derive cost-effectiveness acceptability frontiers (CEAFs), which plot the probability of the optimal strategy being cost-effective across a range of values of willingness-to-pay per additional QALY [54, 55]. Analyses were undertaken in Stata version 12MP.

## Results

### Participants and physical activity during the trial

In total, 261 (74%) of the eligible women completed the baseline questionnaires and provided written informed consent. Approximately, 7% and 15% of the study population was lost to follow-up at 6 and 12 months respectively. Women had a mean age of 52 years, with 90% of them being of white ethnicity, and 67% classified as overweight or obese. Most women (70%) were postmenopausal, and were experiencing approximately 9 moderately troublesome vasomotor menopausal symptoms a day. At 6 months, the *Exercise-Social support* and *Exercise-DVD* groups were significantly more engaged in vigorous physical activity per week relative to the control group at the 5% (*p-value* = 0.03) and 10% (*p-value* = 0.08) levels respectively. These improvements were not sustained until the 12 months follow-up, where women in the control group reported participating in slightly more vigorous exercise on average per week. For moderate levels of physical activity, differences between the two comparator groups were marginal. More detailed information about the trial participants and findings can be found elsewhere [20].

### Resource use and costs

Mean per woman resource use for the three groups of the trial and for both follow-up periods was particularly low. The most frequent contacts from a NHS/PSS perspective were with the GP and practice nurse. From a societal perspective, women mostly reported time lost from unpaid work and a low level of out-of-pocket expenses. These are shown in S1 Table.

The mean per woman cost for developing and delivering the *Exercise-Social support* and *Exercise-DVD* interventions was £37 and £78 respectively. None of the other cost components included in the NHS/PSS and societal perspectives was significantly different between the two exercise interventions and the control group. A disaggregated list of mean per woman costs at 6 and 12 months follow-up is provided at S2 and S3 Tables respectively.

In terms of total NHS/PSS costs, the *Exercise-Social support* group was £52 (CIs: £18 to £86) and £18 (CIs: -£68 to £105) more expensive per woman than the control group at 6 and 12 months follow-up. For these two follow-up periods, the *Exercise-DVD* group costed £61 (CIs: £41 to £82) and £27 (CIs: -£33 to £87) more per woman than the control group. In terms of total societal costs, both exercise groups were more expensive than the control group at 6 months, with cost difference being of similar magnitude to that of the NHS/PSS costs. At 12 months, however, the total societal cost of both the *Exercise-Social support* and the *Exercise-DVD* groups were £35 (CIs: -£174 to £104) and £16 (CIs: -£133 to £101) less per woman than the control group respectively. The mean per woman total costs for each intervention, perspective, and follow-up period are shown in Table 2 (Imputed analysis) and S4 Table (Complete-case analysis).

**Table 2:** Mean per-woman total costs and outcomes—Multiple imputation analysis (£, 2013/14 prices).

Costs and outcomes	Control group (n = 87)		Exercise—Social support (n = 87)		Exercise—DVD (n = 87)		Difference (Exercise—Social support vs. Control group)			Difference (Exercise—DVD vs. Control group)		
	Raw Mean	SE	Raw Mean	SE	Raw Mean	SE	Adjusted Mean§	95% CIs*		Adjusted Mean§	95% CIs*	
***Baseline quality of life***												
SF-6D	0.704	0.014	0.694	0.014	0.662	0.014	**-0.010**	-0.049	0.029	**-0.042**	-0.081	-0.003
***6 months***												
NHS/PSS perspective	32	9	80	14	92	6	**52**	18	86	**61**	41	82
Societal perspective	41	10	95	19	100	8	**59**	14	103	**60**	35	86
Quality-adjusted life-years (QALYs)	0.355	0.006	0.353	0.006	0.334	0.006	**0.006**	-0.002	0.014	**-0.003**	-0.010	0.005
***12 months***												
NHS/PSS perspective	84	25	96	29	108	15	**18**	-68	105	**27**	-33	87
Societal perspective	178	57	125	35	151	24	**-35**	-174	104	**-16**	-133	101
Quality-adjusted life-years (QALYs)	0.714	0.013	0.713	0.013	0.675	0.013	**0.013**	-0.010	0.036	**-0.002**	-0.025	0.020

* Obtained with robust standard errors based on Huber-White sandwich estimators.

§ Costs were adjusted for age, BMI classification, menopause status, and frequency of symptoms. QALYs were additionally adjusted for baseline quality of life, inpatient stays, employment status, change in the use of antidepressants, and change in symptoms’ frequency.

### Health outcomes

At baseline, mean health-related quality of life for the *Exercise-Social support* and *Exercise-DVD* groups was 0.01 (CIs: -0.049 to 0.029) and 0.042 (CIs: -0.081 to 0.003) less compared with the control group. At 6 months follow-up, the *Exercise-Social support* group had 0.006 (CIs: -0.002 to 0.014) more QALYs gained per woman compared with the control group. Similarly, at 12 months *Exercise-Social support* was more effective, leading to 0.013 (CIs: -0.01 to 0.036) more QALYs gained per woman compared with the control group. The *Exercise-DVD* group had 0.003 (CIs: -0.01 to 0.005) and 0.002 (CIs: -0.025 to 0.02) less QALYs gained per woman compared with the control group. Differences in health outcomes between the two exercise interventions and the control group were small and not statistically significant. The mean per woman outcomes for the imputed and complete-case analyses can are presented in Table 2 and S4 Table respectively.

### Cost-utility analysis

**Fig 1:**
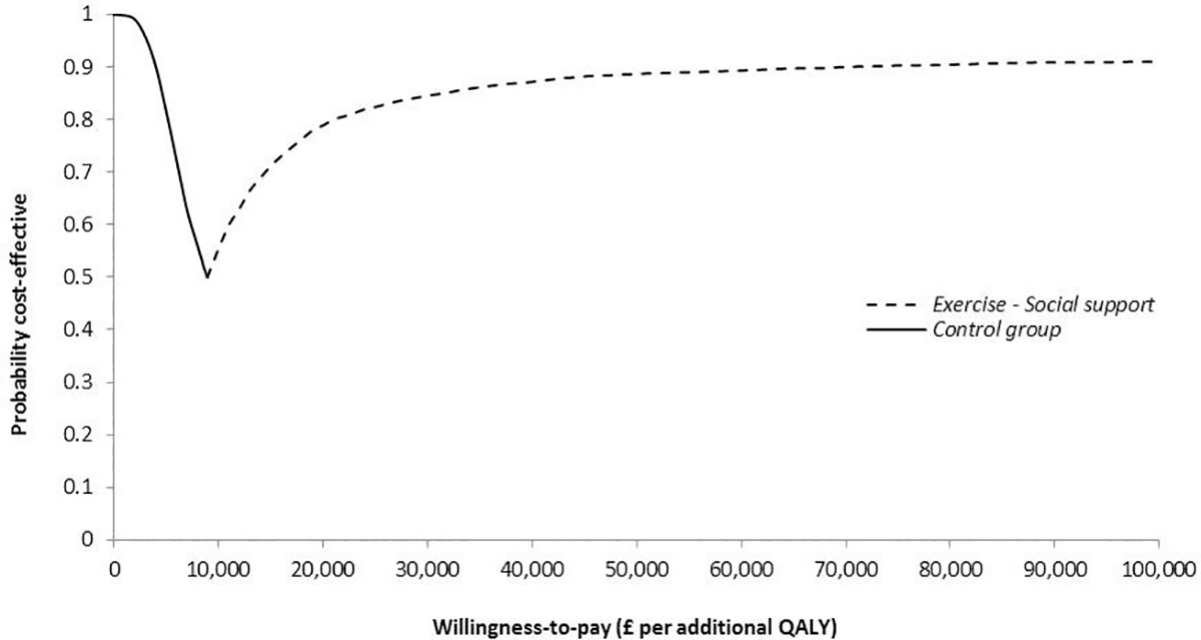
Cost-effectiveness acceptability frontier (CEAF) showing the probability of the optimal intervention being cost-effective at 6 months from a NHS/PSS perspective across different willingness-to-pay values per additional quality-adjusted life-year (QALY).

**Fig 2:**
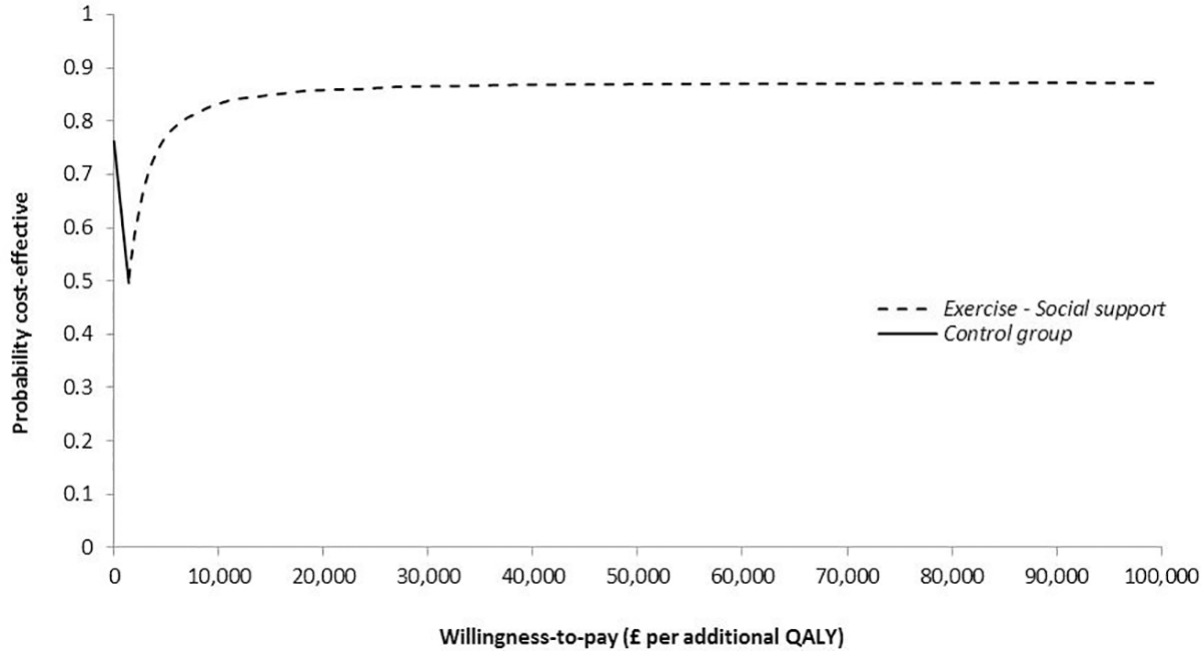
Cost-effectiveness acceptability frontier (CEAF) showing the probability of the optimal intervention being cost-effective at 12 months from a NHS/PSS perspective across different willingness-to-pay values per additional quality-adjusted life-year (QALY).

The *Exercise-DVD* group had the highest cost and lowest QALY gain per woman compared with the other two groups for both follow-up periods, and, therefore, was dominated. For this reason, this intervention was excluded from further analysis. From a NHS/PSS perspective, the *Exercise-Social support* group had an additional 0.006 QALYs gained per woman compared with the control group at 6 months follow-up. Given the additional cost of £52 per woman, the mean ICER for the *Exercise-Social support* group compared with the control group was estimated at £8,940 per QALY. As shown in the CEAF of Fig 1, at the commonly cited willingness-to-pay range of £20,000-£30,000 per QALY [56], *Exercise-Social support* was the optimal intervention at 6 months follow-up with 79%-85% probability of being cost-effective. At 12 months, *Exercise-Social support* had an ICER of £1,413 per QALY compared with the control group, and 86%-87% probability of being cost-effective (Fig 2).

From a societal perspective, the ICER of *Exercise-Social support* compared with the control group at 6 months follow-up was £10,125 per QALY, with *Exercise-Social support* having 76%-83% probability of being cost-effective at the conventionally acceptable willingness-to-pay range of £20,000-£30,000 per QALY (S1 Fig). At 12 months follow-up, *Exercise-Social support* was the least expensive and more effective (dominating) intervention, with nearly 90% probability of being cost-effective at the different values of willingness-to-pay per additional QALY (S2 Fig). The complete-case analysis led to very similar cost-effectiveness point-estimates.

## Discussion

This study assessed the cost-utility of *Exercise-Social support* and *Exercise-DVD* interventions relative to a control group for women with vasomotor menopausal symptoms. During the 12 months of the trial, *Exercise-Social support* led to a small gain in health-related quality of life compared with the control group in the context of many other broader wellbeing benefits of exercise that may not be captured by a health measure like the SF-12. Such examples are sleep quality and family participation in aerobic exercise, that were evident in the trial [20], or general self-esteem, sense of achievement, and life satisfaction that are commonly linked with physical activity in postmenopausal women and general adult or older population [57–60]. Given the small incremental costs involved, it is very likely that *Exercise-Social support* is a cost-effective intervention. This is supported by the bootstrapping results, which showed that at the end of the trial’s period, *Exercise-Social support* was found to be the optimal intervention with 86% (NHS/PSS perspective) and 90% (Societal perspective) probability of being cost-effective at current acceptable thresholds of willingness-to-pay per additional QALY in the UK. This may reflect the findings of the clinical study [20], which showed that the *Exercise-Social support* group had significantly lower somatic symptoms and sleep problems, as well as lower anxiety and depression scores, which were clinically meaningful, compared with the control group.

However, the *Exercise-DVD* intervention did not lead to better health and economic benefits compared with the control group, despite that both *Exercise-Social support* and *Exercise-DVD* groups reported participating in more vigorous intensity exercise [20]. This may indicate that exercise per se does not directly impact on vasomotor menopausal symptoms. A possible explanation is that the social component of the *Exercise-Social support* therapy drives its cost-effectiveness. Social influences have been shown to act as powerful nudges through information sharing and peer pressure [61, 62]. Evidence on the impact of social support on health and wellbeing are available in the literature [63], and these can even reach the more disadvantaged population groups [64]. Social influences were inherent to the *Exercise-Social support* therapy, and may in turn have provided quality of life gains at a very low cost.

While this study concludes that only social versions of exercise interventions are likely to be cost-effective in the management of vasomotor menopausal symptoms, this finding may not be fully in line with the evidence provided from the only other economic evaluation of exercise in this context. That study used a 6 month time horizon and concluded that four unsupervised sessions of physical activity per week, with each lasting more than 50 minutes, is a cost-effective intervention [21]. Although it is not possible to assess how the unsupervised sessions were carried out, any potential difference in the conclusions may be attributed to the fact that the study was based on a sample of women with relatively low frequency of vasomotor symptoms whereas Active Women trial recruited women experiencing a substantial number of vasomotor menopausal symptoms. Nevertheless, given the small costs associated with exercise interventions, evidence suggests that these are commonly found to be cost-effective across different population groups [65–69].

Our study benefited from data from one of the largest randomised controlled trials to date on the clinical effectiveness of exercise in this population group [20], with women being recruited from 23 general practices across West Midlands in the UK. The cost-effectiveness analysis was carried out in line with the recommended practices for economic evaluation and utilised appropriate statistical techniques [70]. National sources were used for costing purposes, and outcome data were obtained from a widely applied preference-based health-related quality of life measure. These, in addition to the fact that cost-effectiveness was explored from both a NHS/PSS and a societal perspective and for both 6 and 12 months follow-up, is likely to have extended the transferability of the study’s findings beyond the UK setting.

The study, however, has limitations. First, there is a potential self-selection bias of people in exercise interventions, which often reduces the difference in the overall benefits observed because non-intervention groups are also undertaking healthy behaviours. Second, women in the study were asked to recall resource use over the past six months, which, despite being an efficient approach, is likely to have introduced a recall bias into the study. Third, vasomotor menopausal symptoms commonly impact on broader aspects of wellbeing than health status [7, 71]. It is, therefore, likely that the broader benefits of exercise on women’s wellbeing may have been inadequately captured. Outcome measures of broader wellbeing, such as the ICECAP-A [72], which has been found to have good psychometric properties in the contexts of women’s health and mental health [73, 74], may have been more appropriate in the cost-effectiveness assessment of exercise for vasomotor menopausal symptoms. Fourth, the human capital approach tends to overestimate productivity losses [75, 76]. Nevertheless, the cost-effectiveness estimates produced from the two perspectives of analysis were very similar. Fifth, multiple imputation relies on the assumption that missing data are independent of unobserved variables, and therefore, are missing at random. This was perceived as a fair assumption, and it is unlikely that there is a systematic bias between trial-arms, which further supports the validity of the study’s results. Finally, the trial’s period allowed only the short-term impacts of exercise to be seen. Considering the additional long-term health benefits associated with exercise [77, 78], in an ageing population, *Exercise-Social support* therapy may offer even greater value for the increasingly limited public health care resources in a longer time horizon.

## Conclusions

Currently, although the Scientific Advisory Committee of the Royal College of Obstetricians and Gynaecologists in the UK and their Patient Information Committee favour sustained aerobic exercise for vasomotor menopausal symptoms [16, 17], evidence for its clinical effectiveness is lacking. This study is the first to bring the cost-effectiveness argument into the policy and clinical debate. The study explored the cost-effectiveness of an individual and social support-based exercise interventions for women with vasomotor menopausal symptoms, and the findings indicated that exercise is very likely to offer value for money when it is accompanied by exercise social support groups in the local community. Although the direct benefit of this intervention on health-related quality of life was found to be small, the marginal additional costs involved in a context where there are broader wellbeing and long-terms health benefits associated with exercise and social participation, suggests that social versions of exercise interventions should be further explored in the management of vasomotor menopausal symptoms.

## Supporting information

S1 Table Mean per-woman resource use by intervention arm during the trial period. (PDF)

S2 Table Disaggregated mean per-woman costs at 6 months follow-up (£, 2013/14 prices). (PDF)

S3 Table Disaggregated mean per-woman costs at 12 months follow-up (£, 2013/14 prices). (PDF)

S4 Table Mean per-woman total costs and outcomes—Complete-case analysis (£, 2013/14 prices). (PDF)

S1 Fig Cost-effectiveness acceptability frontier (CEAF) showing the probability of the optimal intervention being cost-effective at 6 months from a societal perspective across different willingness-to-pay values per additional quality-adjusted life-year (QALY). (TIF)

S2 Fig Cost-effectiveness acceptability frontier (CEAF) showing the probability of the optimal intervention being cost-effective at 12 months from a societal perspective across different willingness-to-pay values per additional quality-adjusted life-year (QALY). (TIF)
