# Supplementary material for: Aerobic exercise for vasomotor menopausal symptoms: A cost-utility analysis based on the Active Women trial
Source: PLoS One. 2017 Sep 26;12(9):e0184328. doi: 10.1371/journal.pone.0184328 (PMC5614527; doi:10.1371/journal.pone.0184328)
Supplement: S1 Table — (PDF) [file pone.0184328.s001.pdf]

**S1 Table. Mean per-woman resource use by intervention arm during the trial period**

| Resource use / Follow-up periods | 6 months follow-up | | | | | | 12 months follow-up | | | | | |
|---|---|---|---|---|---|---|---|---|---|---|---|---|
| | Control group | | Exercise — Social support | | Exercise — DVD | | Control group | | Exercise — Social support | | Exercise — DVD | |
| | Mean | SD | Mean | SD | Mean | SD | Mean | SD | Mean | SD | Mean | SD |
| ***NHS/PSS perspective*** | | | | | | | | | | | | |
| GP (surgery) | 0.39 | 1.00 | 0.36 | 1.07 | 0.17 | 0.77 | 0.59 | 2.48 | 0.17 | 0.49 | 0.19 | 0.79 |
| GP (telephone) | 0.00 | 0.00 | 0.00 | 0.00 | 0.00 | 0.00 | 0.03 | 0.24 | 0.00 | 0.00 | 0.00 | 0.00 |
| Nurse (surgery) | 0.06 | 0.36 | 0.04 | 0.25 | 0.01 | 0.11 | 0.06 | 0.38 | 0.00 | 0.00 | 0.04 | 0.27 |
| Gynaecologist (or other hospital doctor) | 0.00 | 0.00 | 0.03 | 0.16 | 0.00 | 0.00 | 0.04 | 0.27 | 0.00 | 0.00 | 0.01 | 0.12 |
| Psychologist (or counsellor) | 0.00 | 0.00 | 0.08 | 0.68 | 0.00 | 0.00 | 0.00 | 0.00 | 0.00 | 0.00 | 0.00 | 0.00 |
| Repeat prescription | 0.00 | 0.00 | 0.21 | 0.99 | 0.06 | 0.37 | 0.07 | 0.40 | 0.20 | 0.93 | 0.11 | 0.73 |
| Free prescription | 0.07 | 0.26 | 0.04 | 0.20 | 0.04 | 0.19 | 0.03 | 0.17 | 0.00 | 0.00 | 0.01 | 0.12 |
| ***Additional resource use for societal perspective*** | | | | | | | | | | | | |
| Private therapist | 0.02 | 0.19 | 0.07 | 0.41 | 0.00 | 0.00 | 0.03 | 0.24 | 0.00 | 0.00 | 0.07 | 0.43 |
| Days of paid work lost | 0.00 | 0.00 | 0.01 | 0.11 | 0.00 | 0.00 | 0.36 | 2.99 | 0.00 | 0.00 | 0.30 | 1.66 |
| Unpaid hours lost per week | 0.20 | 1.42 | 0.22 | 1.07 | 0.73 | 3.18 | 0.44 | 1.71 | 0.08 | 0.37 | 0.16 | 0.69 |
| Out-of-pocket payments (prescriptions) | 0.04 | 0.19 | 0.05 | 0.23 | 0.04 | 0.19 | 0.05 | 0.21 | 0.06 | 0.25 | 0.01 | 0.12 |
| Out-of-pocket payments (non-prescriptions) | 0.10 | 0.30 | 0.05 | 0.23 | 0.01 | 0.11 | 0.13 | 0.34 | 0.02 | 0.13 | 0.04 | 0.21 |
