# Supplementary material for: Aerobic exercise for vasomotor menopausal symptoms: A cost-utility analysis based on the Active Women trial
Source: PLoS One. 2017 Sep 26;12(9):e0184328. doi: 10.1371/journal.pone.0184328 (PMC5614527; doi:10.1371/journal.pone.0184328)
Supplement: S2 Table — (PDF) [file pone.0184328.s002.pdf]

S2 Table. Disaggregated mean per-woman costs at 6 months follow-up (£, 2013/14 prices)

Interventions / Costing perspectives	Control group		Exercise — Social support		Exercise — DVD		Difference (Exercise — Social support vs. Control group)			Difference (Exercise — DVD vs. Control group)		
	Mean	SD	Mean	SD	Mean	SD	**Mean**	95% CIs*		**Mean**	95% CIs*	
NHS/PSS perspective												
Intervention	0.00	0.00	36.75	0.00	78.09	0.00	36.75	–	–	78.09	–	–
GP (surgery)	25.42	65.80	23.98	70.87	11.40	50.85	-1.44	-22.05	19.16	-14.02	-31.76	3.71
Nurse (surgery)	0.81	4.85	0.52	3.41	0.17	1.49	-0.29	-1.55	0.98	-0.64	-1.69	0.40
Gynaecologist (or other hospital doctor)	0.00	0.00	3.48	21.45	0.00	0.00	3.48	-0.67	7.63	0.00	0.00	0.00
Psychologist (or counsellor)	0.00	0.00	8.70	76.35	0.00	0.00	8.70	-4.46	21.86	0.00	0.00	0.00
Repeat prescription	0.00	0.00	3.85	18.38	1.14	6.78	3.85	-0.27	7.97	1.14	-0.35	2.64
Free prescription	5.65	31.70	1.56	7.81	1.39	7.18	-4.09	-10.95	2.77	-4.26	-11.06	2.54
Additional resource use for societal perspective												
Private therapist	0.00	0.00	3.68	23.49	0.00	0.00	3.68	-0.94	8.31	0.00	0.00	0.00
Days of paid work lost	0.00	0.00	1.11	9.77	0.00	0.00	1.11	-0.69	2.92	0.00	0.00	0.00
Unpaid hours lost per week	1.70	11.79	1.83	8.89	6.02	26.41	0.13	-3.17	3.44	4.32	-1.93	10.57
Out-of-pocket payments (prescriptions)	1.18	6.64	1.21	6.61	0.20	1.17	0.04	-2.04	2.12	-0.97	-2.42	0.47
Out-of-pocket payments (non-prescriptions)	2.52	8.88	1.25	5.59	0.06	0.55	-1.27	-3.64	1.10	-2.46	-4.46	-0.46

* Obtained with 1,000 bias-corrected and accelerated (BCa) bootstrap resamples.
