# Supplementary material for: Aerobic exercise for vasomotor menopausal symptoms: A cost-utility analysis based on the Active Women trial
Source: PLoS One. 2017 Sep 26;12(9):e0184328. doi: 10.1371/journal.pone.0184328 (PMC5614527; doi:10.1371/journal.pone.0184328)
Supplement: S3 Table — (PDF) [file pone.0184328.s003.pdf]

S3 Table. Disaggregated mean per-woman costs at 12 months follow-up (£, 2013/14 prices)

**Costing perspectives** / **Interventions**	Control group		Exercise — Social support		Exercise — DVD		Difference (Exercise — Social support vs. Control group)			Difference (Exercise — DVD vs. Control group)		
	Mean	SD	Mean	SD	Mean	SD	**Mean**	95% CIs*		**Mean**	95% CIs*	
NHS/PSS perspective												
GP (surgery)	39.18	163.2	11.33	32.29	12.24	51.80	-27.85	-66.01	10.32	-26.93	-65.68	11.82
GP (telephone)	0.78	6.50	0.00	0.00	0.00	0.00	-0.78	-2.11	0.54	-0.78	-2.11	0.54
Nurse (surgery)	0.78	5.09	0.00	0.00	0.58	3.57	-0.78	-2.02	0.46	-0.20	-1.72	1.32
Gynaecologist (or other hospital doctor)	5.83	35.86	0.00	0.00	1.91	16.02	-5.83	-14.35	2.69	-3.91	-13.47	5.65
Repeat prescription	1.34	7.33	3.77	17.22	2.12	13.59	2.42	-2.01	6.85	0.78	-3.05	4.60
Free prescription	6.96	28.66	1.21	6.72	0.63	5.26	-5.75	-12.43	0.93	-6.33	-13.01	0.35
Additional resource use for societal perspective												
Private therapist	1.57	13.15	0.00	0.00	1.43	11.95	-1.57	-4.35	1.21	-0.14	-4.36	4.07
Days of paid work lost	30.62	256.16	0.00	0.00	24.67	136.72	-30.62	-90.67	29.44	-5.94	-73.72	61.83
Unpaid hours lost per week	3.68	14.18	0.65	3.07	1.30	5.76	-3.03	-6.46	0.41	-2.37	-5.94	1.20
Out-of-pocket payments (prescriptions)	0.71	4.11	2.18	10.17	0.22	1.81	1.48	-1.18	4.13	-0.49	-1.57	0.59
Out-of-pocket payments (non-prescriptions)	3.56	12.58	0.25	2.00	0.28	1.56	-3.31	-6.26	-0.35	-3.28	-6.21	-0.34

* Obtained with 1,000 bias-corrected and accelerated (BCa) bootstrap resamples.
