# Supplementary material for: Aerobic exercise for vasomotor menopausal symptoms: A cost-utility analysis based on the Active Women trial
Source: PLoS One. 2017 Sep 26;12(9):e0184328. doi: 10.1371/journal.pone.0184328 (PMC5614527; doi:10.1371/journal.pone.0184328)
Supplement: S4 Table — (PDF) [file pone.0184328.s004.pdf]

S4 Table. Mean per-woman total costs and outcomes—Complete-case analysis (£, 2013/14 prices)

Costs and outcomes \ Interventions	Control group		Exercise — Social support		Exercise — DVD		Difference (Exercise — Social support vs. Control group)			Difference (Exercise — DVD vs. Control group)		
	Raw Mean	SE	Raw Mean	SE	Raw Mean	SE	**Adjusted Mean§**	95% CIs*		**Adjusted Mean§**	95% CIs*	
6 months												
NHS/PSS perspective	32	10	79	10	92	10	**50**	16	84	**61**	40	82
Societal perspective	41	13	93	13	100	13	**56**	13	100	**60**	34	85
Quality-adjusted life-years (QALYs)	0.356	0.007	0.355	0.007	0.335	0.007	**0.006**	-0.002	0.014	**-0.003**	-0.010	0.005
12months												
NHS/PSS perspective	84	20	84	21	109	20	**2**	-66	70	**25**	-37	87
Societal perspective	144	34	108	35	158	34	**-26**	-127	74	**19**	-84	122
Quality-adjusted life-years (QALYs)	0.701	0.015	0.708	0.015	0.658	0.014	**0.013**	-0.011	0.037	**-0.004**	-0.026	0.018

* Obtained with 1,000 bias-corrected and accelerated (BCa) bootstrap resamples.

§ Costs were adjusted for age, BMI classification, menopause status, and frequency of symptoms. QALYs were additionally adjusted for baseline quality of life, inpatient stays, employment status, change in the use of antidepressants, and change in symptoms' frequency.
