# Supplementary figures and images for: Aerobic exercise for vasomotor menopausal symptoms: A cost-utility analysis based on the Active Women trial
Source: PLoS One. 2017 Sep 26;12(9):e0184328. doi: 10.1371/journal.pone.0184328 (PMC5614527; doi:10.1371/journal.pone.0184328)

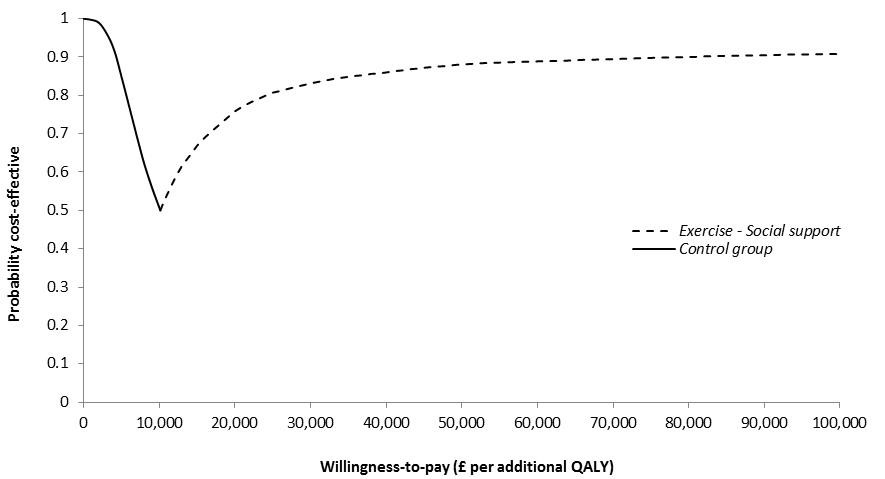

Supplement: S1 Fig — (TIF) [file pone.0184328.s005.tif]

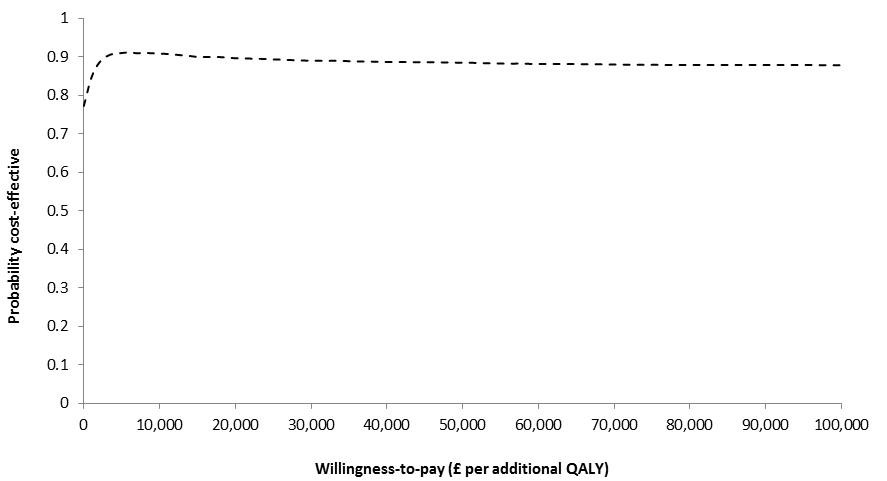

Supplement: S2 Fig — (TIF) [file pone.0184328.s006.tif]
